# Glioblastoma and Blood Microenvironment Predictive Model for Life Expectancy of Patients

**DOI:** 10.3390/biomedicines13051040

**Published:** 2025-04-25

**Authors:** Alexander N. Chernov, Sofia S. Skliar, Mikalai M. Yatskou, Victor V. Skakun, Sarng S. Pyurveev, Ekaterina G. Batotsyrenova, Sergey N. Zheregelya, Guodong Liu, Vadim A. Kashuro, Dmitry O. Ivanov, Sergey D. Ivanov

**Affiliations:** 1 Biological Chemistry Department, Federal State Budgetary Educational Institution of Higher Education Saint Petersburg State Pediatric Medical University of the Ministry of Health of Russia, 194100 Saint Petersburg, Russia; dr.purveev@gmail.com (S.S.P.); bkaterina2009@yandex.ru (E.G.B.); sgerege-ly@bk.ru (S.N.Z.); kashuro@yandex.ru (V.A.K.); doivanov@yandex.ru (D.O.I.); 2 Department of General Pathology and Pathophysiology, Federal State Budgetary Institution of Science “Institute of Experimental Medicine”, 197022 Saint Petersburg, Russia; 3 Laboratory of Neurooncology of Polenov Neurosurgical Institute, Almazov National Medical Research Centre, 197341 Saint Petersburg, Russia; sklyar_ss@almazovcentre.ru; 4 Department of System Analysis and Computer Modeling, Belarussian State University, 220030 Minsk, Belarus; yatskou@bsu.by (M.M.Y.); skakun@bsu.by (V.V.S.); 5 Department of Neurosurgery, The Second Affiliated Hospital of Chongqing Medical University, Chongqing 400016, China; 304678@hospital.cqmu.edu.cn; 6 Department of Maxillofacial Surgery and Surgical Dentistry, Medical Institute of Saint Petersburg State University, 199034 Saint Petersburg, Russia; 7 Department of Anatomy and Physiology of Humans and Animals, Herzen State Pedagogical University of Russia, 191186 Saint Petersburg, Russia; 8 Federal State Budgetary Institution “National Medical Research Center of Oncology named after N.N. Petrov” of the Ministry of Health of the Russian Federation, 197758 Saint Petersburg, Russia; sdivanov44@mail.ru

**Keywords:** glioblastoma, predictive model, chemotherapy, blood cells, blood proteins, tumor microenvironment, carboplatin, band neutrophils, total protein, life expectancy of patients

## Abstract

**Background:** Glioblastoma multiforme (GBM) is a very malignant brain tumor. GBM exhibits cellular and molecular heterogeneity that can be exploited to improve patient outcomes by individually tailoring chemotherapy regimens. **Objective:** Our objective was to develop a predictive model of the life expectancy of GBM patients using data on tumor cells’ sensitivity to chemotherapy drugs, as well as the levels of blood cells and proteins forming the tumor microenvironment. **Methods:** The investigation included 31 GBM patients from the Almazov Medical Research Centre (Saint Petersburg, Russia). The cytotoxic effects of chemotherapy drugs on GBM cells were studied by an MTT test using a 50% inhibitory concentration (IC_50_). We analyzed the data with life expectancy by a one-way ANOVA, principal component analysis (PCA), ROC, and Kaplan–Meier survival tests using GraphPad Prism and Statistica 10 software. **Results:** We determined in vitro the IC_50_ of six chemotherapy drugs for GBM and 32 clinical and biochemical blood indicators for these patients. This model includes an assessment of only three parameters: IC_50_ of tumor cells to carboplatin (CARB) higher than 4.115 μg/mL, as well as levels of band neutrophils (NEUT-B) below 2.5% and total protein (TP) above 64.5 g/L in the blood analysis, which allows predicting with 83.3% probability (sensitivity) the life expectancy of patients for 15 months or more. In opposite, a change in these parameters—CARB above 4115 μg/mL, NEUT-B below 2.5%, and TP above 64.5 g/L—predict with 83.3% probability (specificity) no survival rate of GBM patients for more than 15 months. The relative risk for CARB was 6.41 (95 CI: 4.37–8.47, *p* = 0.01); for NEUT-B, the RR was 0.40 (95 CI: 0.26–0.87, *p* = 0.09); and for TP, it was 2.88 (95 CI: 1.57–4.19, *p* = 0.09). Overall, the model predicted the risk of developing a positive event (an outcome with a life expectancy more than 10 months) eight times (95 CI 6.34–9.66, *p* < 0.01). Cross k-means validation on three clusters (n = 10) of the model showed that its average accuracy (sensitivity and specificity) for cluster 1 was 74.98%; for cluster 2, it was 66.7%; and for cluster 3, it was 60.0%. At the same time, the differences between clusters 1, 2, and 3 were not significant. The results of the Sobel test show that there are no interactions between the components of the model, and each component is an independent factor influencing the event (life expectancy, survival) of GBM patients. **Conclusions:** A simple predictive model for GBM patients’ life expectancy has been developed using statistical analysis methods.

## 1. Introduction

Glioblastoma multiforme (GBM) is a highly malignant tumor in the central nervous system with median untreated patients’ survival of three months [1]. To date, for many cases, reliable reasons for GBM occurrence and development have not been clearly established. Possible risk factors for GBM development include age [2], hereditary tumor history [3], de novo germ line mutations [4], and exposure to ionizing [5] and non-ionizing electromagnetic radiation of cellular phones [6]. Factors that contribute to GBM development and hinder the treatment of patients include metabolic heterogeneity of tumor cells, among which there are cells with an increased rate of oxygen metabolism, glucose, proliferation, and dormant cells [7]; a hypoxic tumor microenvironment, which is a factor for the induction of epithelial–mesenchymal transition and tumor cell resistance [8]; the development of multiple GBM cell radio- and chemoresistance associated with the presence of mutations in driver and suppressor genes, as well as with epigenetic modifications [9]. Given the short survival term after diagnosis, the timing of testing for personalized treatment regimens and including the entire prediction procedure, as well as the availability for translation of this technology when it is introduced into clinical practice, are critical.

Currently used treatment protocols for GBM patients include surgical resection of the maximum possible neoplasm volume (49%) followed by radiochemotherapy with temozolomide (TMZ) (11%) [10]. The use of radiation diagnostics (computer and magnetic resonance imaging) has made it possible to increase the accuracy and adequacy of ionizing radiation energy delivery to the tumor, while minimizing the irradiation of surrounding tissues and organs [11]. However, the median of life expectancy in GBM patients treated with radiotherapy and chemotherapy is still only about 12–15 months [1]. If GBM cells develop resistance to TMZ, other chemotherapy drugs are used in clinical conditions, such as doxorubicin (DOX), carboplatin (CARB), cisplatin (CIS), and etoposide (ETO), which have different intracellular molecular mechanisms [12,13,14].

To date, more than 160 growth factors have been identified [15]. Of the growth factors and neurotrophins, nerve growth factor (NGF) is of the greatest interest for the therapy of diseases of the nervous system. NGF promotes the survival, maintenance, and differentiation of sympathetic cholinergic neurons and glial cells through the tyrosine kinase A (TrkA) receptor interaction [16]. At the same time, NGF, binding to the p75 receptor, induces cell apoptosis [17]. Antitumor effects of NGF have also been established in relation to some types of tumors. For example, such effects include NGF inhibition of angiogenesis and neuroblastoma invasion [18]. Expression of the TrkA receptor on neuroblastoma cells is a favorable prognostic marker of the outcome of the disease and a prerequisite for spontaneous tumor regression [19]. Alteration of TrkA/p75 receptors on PC12 pheochromocytoma cells determines their sensitivity to DOX and CIS [20]. Since NGF is also secreted by blood cells and nerve cells [21] that form the tumor microenvironment, it has been suggested that NGF may influence the sensitivity of GBM cells to chemotherapy and thus the survival of patients.

Taking into account such short life expectancy rates in GBM patients with modern treatment methods, it is necessary to develop new short-term technologies determining biomarkers for predicting therapy effectiveness, which will allow for more successful access to its use in clinical practice.

## 2. Materials and Methods

### 2.1. Patients

This investigation included 31 GBM patients who were treated in the Almazov Medical Research Centre (Saint Petersburg, Russia) in 2021–2024. All patients provided informed consent and underwent MRIs, tumor resections in the neurology department, and histological study in the pathology section. The investigation was approved by the local Ethics Committee of the Institute of Experimental Medicine (No. 6/20, from 21 October 2020).

### 2.2. Cell Culture

GBM biopsies were taken from all subjects and were cut into little particles under sterile conditions in a laminar box. GBM cells were divided in a 0.25% trypsin-EDTA solution (Sigma-Aldrich, St. Louis, MO, USA) at 37 °C for 5 min and grown at 1 × 10^4^ cells per well in 96-well plates (TPP, Trasadingen, Switzerland) using Dulbecco’s modified Eagle medium (DMEM) with 10% calf serum (Sigma-Aldrich, St. Louis, MO, USA) at 37 °C and 5% CO_2_ for 2 days [22,23].

### 2.3. MTT Analysis

The effects of the chemotherapy and NGF on GBM cells were studied using the MTT assay [24]. We diluted 2–10-fold of chemotherapy drugs and 2-fold of NGF in 50 μL of DMEM into each well of the plate. All drugs’ doses were tested three times. Plates with GBM cells were incubated at 37 °C and 5% CO_2_ for 24 h. The next day, MTT solution (25 μL, 5 mg/mL, Thermo Fisher Scientific Inc., Waltham, MA, USA) was added into the plate wells and incubated for 3 h at 37 °C and 5% CO_2_. Then, 50 μL of isopropanol with 0.04 N HCl was brought into all wells and mixed. The optical density of solution was measured at 540 nm as a test and with 590 nm as the background, using a SpectraMax 250 plate spectrophotometer (Molecular Devices, San Jose, CA, USA). The anticancer effects of the drugs (Table 1) were determined as the percentage of dead cells, based on comparing the optical density of GBM cells wells under positive (100% viable cells) and (empty, 0% viable cells) negative controls by Formula (1):

(1) $$DC(\%)=\frac{OD(control)-OD(test)}{\left(OD(control)-OD(0\%\;VC)\right)}\times 100$$

where DC (%) is the percentage of dead cells; OD (test) is the optical density of the drug at a given dose; and OD (0% VC) is the optical density of wells with a culture medium.

### 2.4. IC_50_ Dose

To investigate the anticancer effects of chemotherapy drugs and NGF, the 50% inhibitory dose (IC_50_) was determined. GBM cells were incubated with NGF, cisplatin (CIS), CARB, doxorubicin (DOX), TMZ, and etoposide (ETO) at various doses, as shown in Table 1.

### 2.5. Reagents

The following reagents were used in the study: Doxorubicin-LANS^®^ (solution of 2 mg/mL, Veropharm, Moscow, Russia); Carboplatin-LANS^®^ (solution of 10 mg/mL, Veropharm, Moscow, Russia); temozolomide (Temodal capsules, 100 mg, Orion Pharma, Espoo, Finland); Cisplatin-LANS^®^ (0.5 mg/mL, Veropharm, Moscow, Russia); etoposide (20 mg/mL, Ebewe Pharma, Unterach am Attersee, Austria); human β-form NGF (10 μg, H9666, Sigma-Aldrich, Inc., St. Louis, MO, USA).

### 2.6. Blood Samples Analysis

Blood samples (3–4 mL) were taken together with tumor surgery samples from all patients with GBM from tumor vessels. Concentrations of blood cells, including basophils (BOs), bound neutrophils (NEUTBs), eosinophils (EOs), immature granulocytes (IGs), metamelocytes (MMCs), myelocytes (MCs), monocytes (MONs), neutrophils (NEUTs), platelets (PLTs), red blood cells (RBCs), segmented neutrophils (NEUTSs), and white blood cells (WBCs); levels of blood proteins, including: albumin (ABN), alanineaminotransferase (ALT), aspartateaminotransferase (AST), C-reactive protein (CRP), hemoglobin (HGB), total bilirubin (TB), and total protein (TP); levels of blood parameters, including: activated partial thromboplastin time (aPTT), hemocrypt (HCT), and glomerular filtration rate (GFR); as well as urea (BUN), glucose (GLU), and creatinine (CREAT) were determined by an Architect c8000 automatic biochemical analyzer (Abbott, Chicago, IL, USA) according to the manufacturer’s instructions [25]. Levels of plateletcrit (PCT), fibrinogen (FBN), and D-dimer (DD) were determined in blood samples by a STA-R Evolution automatic hemostasis analyzer (Stago, France) [26]. We compared the qualitative composition of tumor bed blood and peripheral blood in several patients. The qualitative composition of tumor blood samples and patient peripheral blood samples did not differ significantly.

### 2.7. Predictive Model for Life Expectancy of Glioblastoma Patients

Multiple differences between life expectancy and other parameters were calculated using a one-way ANOVA test. Then, statistically significant parameters were further analyzed using principal component analysis (PCA) using a calculator [27]. To calculate the area under the curve, threshold values, sensitivity, and specificity of predictors, an ROC analysis was completed using the GraphPad Prism program (version 8.01, 09.21.2020, San Diego, CA, USA).

In order to determine the predictive efficiency of chemotherapy drugs, NGF, and blood parameters in GBM patients’ life expectancy, their sensitivity (Se) and specificity (Sp) were calculated [28] as in Formula (2).

(2) $$\mathrm{Se}=\;\frac{a}{\left(a+c\right)}\times 100\%$$

where Se is the sensitivity, a is the true positive result—the number of samples with parameter values above the threshold value in the favorable outcome group of patients; and c is the false negative result—the number of parameter samples with values below the threshold value in the favorable outcome group of patients—as shown in Formula (3):

(3) $$\mathrm{Sp}=\frac{d}{\left(b+d\right)}\times 100\%$$

where Sp is the specificity; b is the false positive result—the number of samples with the level of indicators above the threshold values in the group of fatal outcome patients; d is the true negative result—the number of samples with the level of indicators in the fatal outcome group of patients [28]. Specificity shows the number of true negative results in the patients’ group.

### 2.8. Statistical Analysis

All experiments were conducted in triplicate. The statistical differences between the means of different treatments and their controls were studied by Student’s *t*-test. Data were presented with the standard deviation and were considered significant at *p* < 0.05. To compare the differences between two independent groups with a small number of samples (*n* < 30), the nonparametric Mann–Whitney U-test was used [29]. Descriptive statistics, an ANOVA test, and OS analysis were done by GraphPad Prism, version 8.01, 09.21.2020, San Diego, CA, USA). The relative risk (RR) of the three main predictors and the entire model was calculated using the GIGAcalculator on the website https://www.gigacalculator.com/calculators/odds-ratio-calculator.php (accessed on 2 April 2025). Cross k-means validation and Cox regression analysis were performed using Statistica 10 software (Stat Soft, Tulsa, OK, USA). The Sobel test was calculated using the Sobel test calculator on the website https://www.danielsoper.com/statcalc/calculator.aspx?id=31 (accessed on 2 April 2025) [30].

## 3. Results

The model included clinical and data about the IC_50_ of chemotherapy drugs and NGF for 31 glioblastoma patients, as shown in Table 2 [31].

The data in Table 2 show that our sample of GBM patients included 20 men (64.5%) and 11 women (35.5%) of middle age (59.2 ± 11.7 years, range 31–75 years) with the IDH-1 wild subtype (wt, 83.8%) and Nestin-expressed phenotype (NES, 6.5%). The proliferative activity of GBM cells, measured by the expression of the nuclear antigen Ki-67, was 50–70% for one case (3.2%), 40–50% for one case (3.2%), 35–40% for four tumors (12.9%), 30–35% for one tumor (3.2%), 25–30% for five tumors (16.2%), 18–25% for five tumors (16.2%), 15–20% for three tumors (9.7%), 8–15% for five tumors (16.2%), and 3–5% for one patient’s tumor, respectively. The average life expectancy of patients was 10 ± 6.5 months.

**Figure 1:**
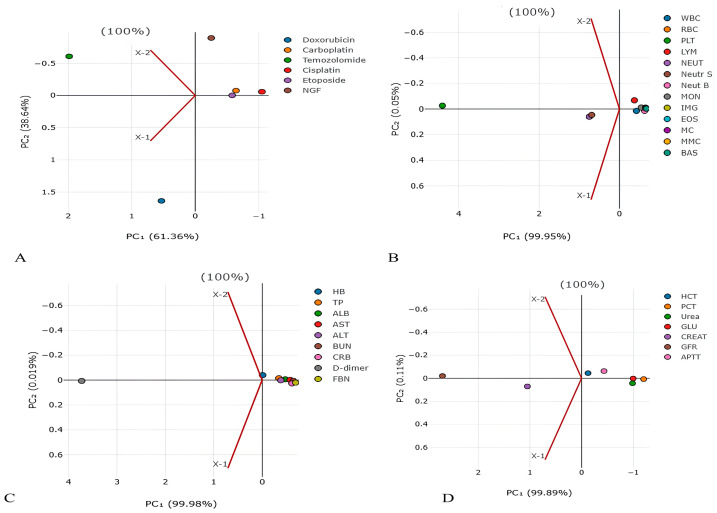
Principal component analysis for detection of main factors from (**A**) chemotherapy and NGF, (**B**) blood cells, (**C**) blood proteins, and (**D**) blood parameters in lifespan of GBM patients. The values on the X and Y axes represent the level of dispersion for each drug. The higher the value, the higher the dispersion (spread) of values for the drug.

**Figure 2:**
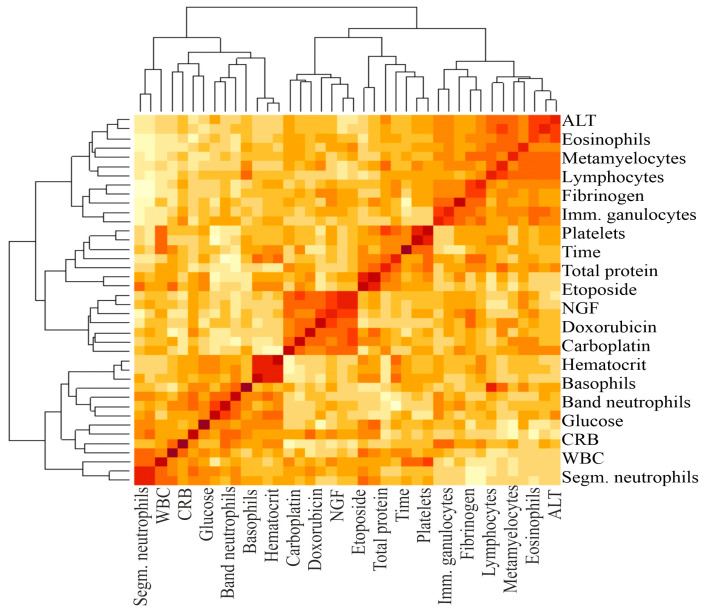
A heat map and a false color image with a dendrogram added to the left side and to the top. The intensity of the color determines the magnitude of the correlation coefficient. The darker the color, the greater the correlation. The brackets at the top and bottom indicate the blood parameters between which the correlation was calculated.

Also, clinical and biochemical blood analysis, as well as hematostasis indicators (D-dimer, fibrinogen), were performed in these patients (Table S1). Since the values of each parameter varied among patients (variance), we used ANOVA to compare them with life expectancy. The one-way ANOVA test allows one to identify the influence of the main factor (life expectancy) on the parameters by comparing their variances. In order to identify possible predictors of life expectancy in GBM patients, one-way ANOVA and PCA were performed (Table 3, Figure 1 and Figure 2).

The results of Table 3 show that when comparing the variances, statistically significant differences with the lifespan were observed for the IC_50_ of CARB, TMZ, CIS, NGF, platelet levels, neutrophils, immature granulocytes, myelocytes, metamyelocytes, eosinophils, basophils, the proteins hemoglobin, albumin, total protein, and D-dimer; and the following parameters: hematocrit, plateletcrit, creatinine, GFR, and APTT.

In order to reduce the number and highlight the most significant parameters influencing life expectancy, the PCA method was used. PCA has identified the following main predictors, shown in Figure 1 and Figure 2: CARB (PC1 2.223124055), NGF (PC1 −1.63257032), platelets (PC1 4.407250456), neutrophils (PC1 0.750175018), D-dimer (PC1 3.729850379), GFR (PC1 2.695131625), creatinine (PC1 1.050472729), and plateletcrit (PC1 −1.202390197).

**Figure 3:**
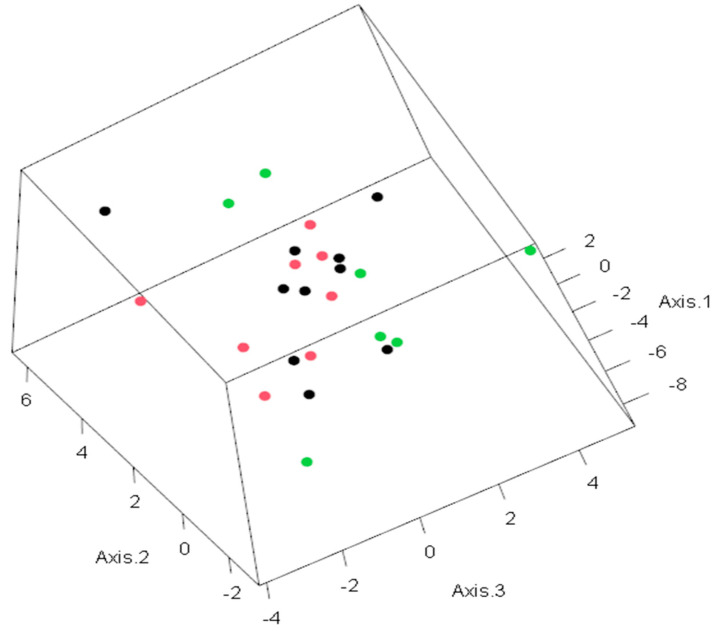
PCA for predictive life expectancy model. The observations in the space of the first three principal components. Colors indicate the following: green—life expectancy higher than 10 months; red—life expectancy less than or equal to 10 months; black—life expectancy unknown. The values on each axis represent the level of dispersion for each patient.

In the next stage, all patients were divided into two groups by life expectancy: group 1 up to 10 months and group 2 more than 10 months (Table 4, Figure 3). To assess the duration of the lifespan of each indicator in GBM patients, their area under the curve (AUC), sensitivity, and specificity were calculated using ROC analysis. Sensitivity allows us to determine the proportion (percentage) of patients whose parameters were above the threshold level in the group with a favorable outcome (survival expectancy of longer than 10 months). Sensitivity shows the number of true positive results in the patients’ group with a favorable outcome. Specificity determines the percentage of patients whose parameters were below the threshold values in the group with a life expectancy shorter than 10 months. Specificity indicates the number of true negative results in a group of patients with an unfavorable outcome. The integral indicator that determines the efficiency or accuracy of a parameter is the AUC.

ROC analysis revealed that band neutrophils, as well as doxorubicin, carboplatin, etoposide, lymphocytes, ALT, AST, albumin, and total protein APTT had an AUC higher than 0.750. Such significant indicators of Table 2 as the IC_50_ of CIS, TMZ, NGF, levels of platelets, immature granulocytes, myelocytes, metamyelocytes, monocytes, eosinophils, basophils, hemoglobin, D-dimer, HCT, PCT, creatinine, and GFR did not have a sufficiently high AUC. In addition, for DOX, CARB, NEUT-B, and TP, the highest likelihood ratio was calculated to be 4.4, 5.25, and 4.286, respectively. This indicator characterizes the ratio of the probability of developing a positive result with a positive outcome to the probability of a positive result with a negative outcome (life expectancy less than 10 months).

**Figure 4:**
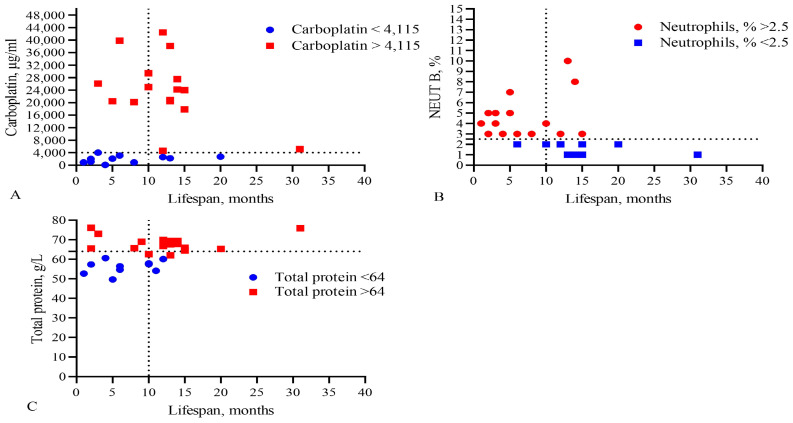
Comparison of levels of (**A**) the IC_50_ of CARB, (**B**) band neutrophils, and (**C**) total protein in the blood with the lifespan of GBM patients. The vertical dotted line separates the groups of patients with low and high life expectancy.

**Figure 5:**
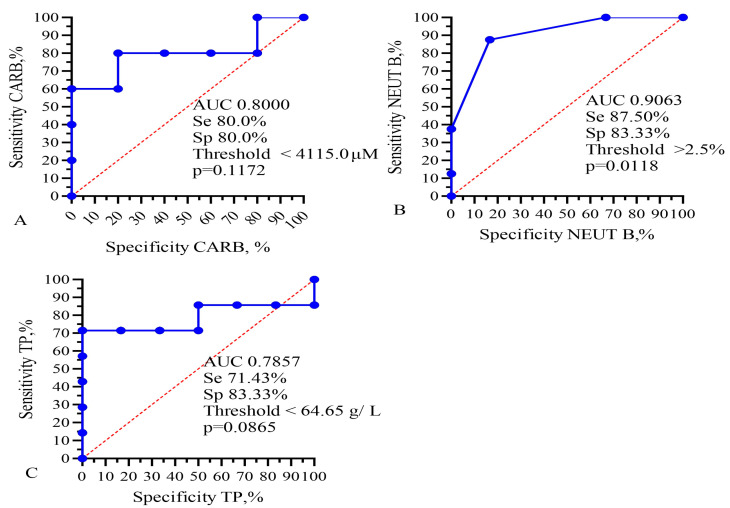
ROC analysis for AUC, sensitivity, and specificity of (**A**) IC_50_ of CARB, (**B**) band neutrophils, and (**C**) total protein for prediction of GBM patients’ lifespan. Se—sensitivity; Sp—specificity.

An analysis of the absolute values of the parameters (by R square) with the life expectancy of patients was carried out (Table 5, Figure 4 and Figure 5).

This analysis showed that only the IC_50_ of DOX, CARB, CIS, and NGF as well as eosinophils, basophils, myelocytes, ALT, AST, total protein, and CRP statistically significant correlated with the life expectancy of GBM patients. In total, the results of Table 2, Table 3 and Table 4 and Figure 1 and Figure 2 allowed us to select three indicators, CARB, NEUT-B, and total protein, as predictors for the life expectancy of GBM patients. For these predictors, ROC curves and graphs of the dependence of their values on life expectancy were constructed, as shown in Figure 4 and Figure 5.

**Figure 6:**
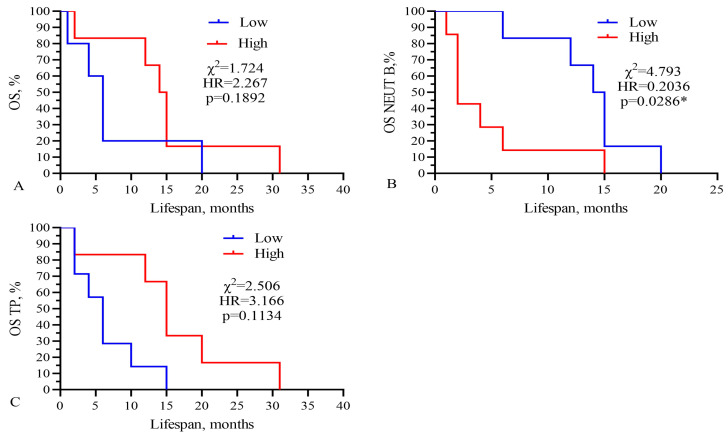
Kaplan–Meier survival analysis for GBM patients based on levels of (**A**) IC_50_ of CARB, (**B**) band neutrophils, and (**C**) total protein. Threshold values: CARB 4115 μg/mL, NEUT-B 2.5%, TP 64.5 g/L. Sign * indicates statistically significant differences at *p* < 0.05.

Finally, Kaplan–Meier survival graphs were created for these four parameters, as shown in Figure 6. Moreover, low levels of band neutrophils (low 2.5%, low 14.5 vs. 2.0 months, χ^2^ = 4.793, HR = 0.2036, *p* = 0.0286) statistically significantly correlated with an increase in the life expectancy of GBM patients.

**Figure 7:**
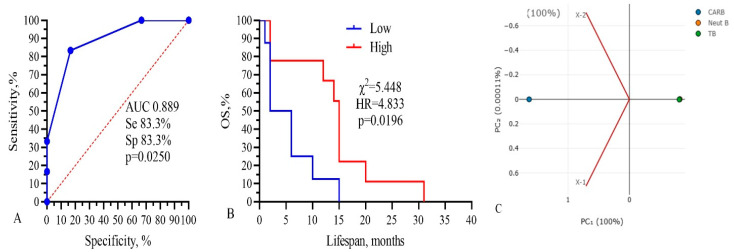
Three-component model, CARB-NEUT-B-TP: (**A**) ROC-curve, (**B**) survival analysis, and (**C**) PCA analysis. Threshold values: CARB 4115 μg/mL, NEUT-B 2.5%, TP 64.5 g/L.

Three from these components (CARB, NEUT-B, TP) were included in the final model, as shown in Figure 7.

The total AUC of the model was 0.889, sensitivity was 83.3%, and specificity was 83.3% (*p* = 0.0250). Kaplan–Meier survival analysis of the model had the following parameters: low 4.0 vs. 15.0 months, χ^2^ =5.448, HR = 4.833, and *p* = 0.0196 (Figure 7B). The PCA standardized value for CARB was 1.154; NEUT-B, −0.58; and TP, 0.57. These data show that only three parameters, IC_50_ of CARB above 4115 μg/mL, NEUT-B below 2.5%, and TP above 64.5 g/L, predict with 83.3% probability (sensitivity) a higher survival rate of GBM patients for more than 15 months. Also, a change in these parameters in the opposite direction, CARB below 4115 μg/mL, NEUT-B above 2.5%, and TP below 64.5 g/L, predict with 83.3% probability (specificity) no survival rate of GBM patients for more than 15 months.

**Figure 8:**
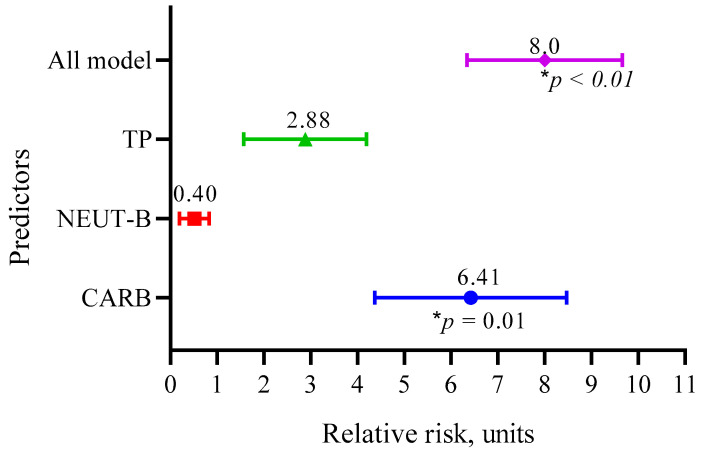
Relative risks of the three main predictors (CARB, NEUT-B, TP) and the whole model.

We also calculated the relative risk of the three main predictors (CARB, NEUT-B, TP) and the whole model, as shown in Figure 8.

The data in Figure 8 show that the RR for CARB was 6.41 (95 CI 4.37–8.47, *p* = 0.01); for NEUT-B, it was 0.40 (95 CI 0.26–0.87, *p* = 0.09); and for TP, it was 2.88 (95 CI 1.57–4.19, *p* = 0.09). Overall, the model predicted the risk of developing a positive event (an outcome with a life expectancy more than 10 months) eight times (95 CI 6.34–9.66, *p* < 0.01).

**Figure 9:**
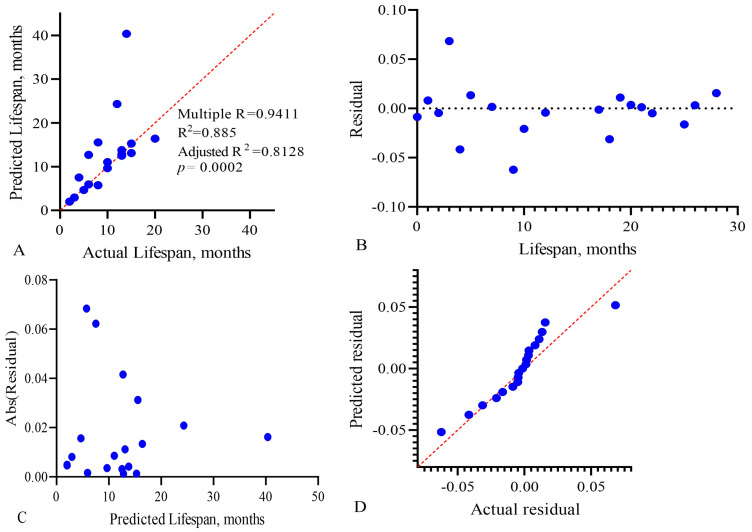
Multivariate linear regression analysis of the predictive model, CARB-NEUT-B-TP, of the life expectancy of GBM patients: (**A**) actual vs. predicted plot, (**B**) residual vs. order plot, (**C**) homoscedasticity plot, and (**D**) QQ plot. Each point on the graphs represents a patient in whom the three factors we studied influenced life expectancy.

To assess the interaction between the three factors of the model and their complex influence on the outcome (lifespan expectancy) of patients, we performed a multivariate regression analysis, demonstrated in Figure 9.

To assess the effectiveness of multiple linear regression, the determination coefficient R^2^ is used. It reflects the degree of dispersion of the result (variance) arising from the contribution of three variables. The value of this coefficient and its sign in the final model show the degree and nature of the relationship between the variables and the outcome. The R^2^ value varies from 0 to 1, and the closer it is to 1, the better the model describes the result. In our model, the adjusted R^2^ value was 0.8128, with *p* = 0.0002, as shown in Figure 9A. To check the adequacy of the constructed multivariate linear regression model, we analyzed the regression residuals (Figure 9B–D). Figure 9C shows the homoscedasticity graph, which estimates the constancy of the vector variance (change in the applied combination) over time. Homoscedasticity shows the homogeneous variability of the values of the variables, expressed in the stability and homogeneity of the variance of the random error of the regression model—the variances are the same at all times of measurement. Since the residual values of the regression model are close to the trend line, this indicates low dispersion of the influence of variables on the outcome over time.

**Figure 10:**
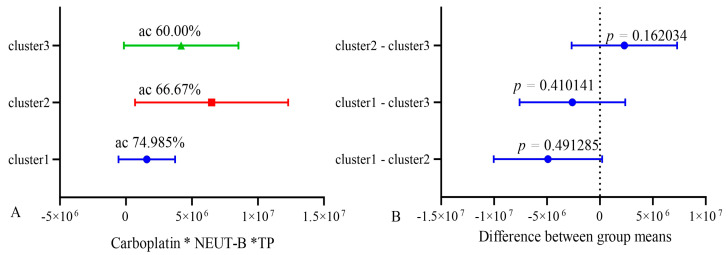
Cross k-means validation between three clusters (n = 10). ac—accuracy.

In order to validate the stability of the model, k-means clustering analysis was used. All patients were divided into three clusters using clustering analysis. Then, the accuracy (sensitivity and specificity) of the model was assessed in groups 1 and 2 and compared with group 3, which served as a reference. After that, the accuracy was assessed in groups 1 and 3, and group 2 was the reference. Also, the accuracy was assessed in groups 2 and 3, and group 1 was used as a control. Cross k-means validation on three clusters (n = 10) of the model showed that its average accuracy (sensitivity and specificity) for cluster 1 was 74.98%; for cluster 2, it was 66.7%; and for cluster 3, was 60.0%. At the same time, the differences between clusters 1, 2, and 3 were not significant, as shown in Figure 10.

These data indicate the relative stability of the model. The lower accuracy in the k-means validation of the model is explained by the smaller number of patients included in each cluster of the model and belonging to the groups of life expectancy below and higher than 10 months.

**Figure 11:**
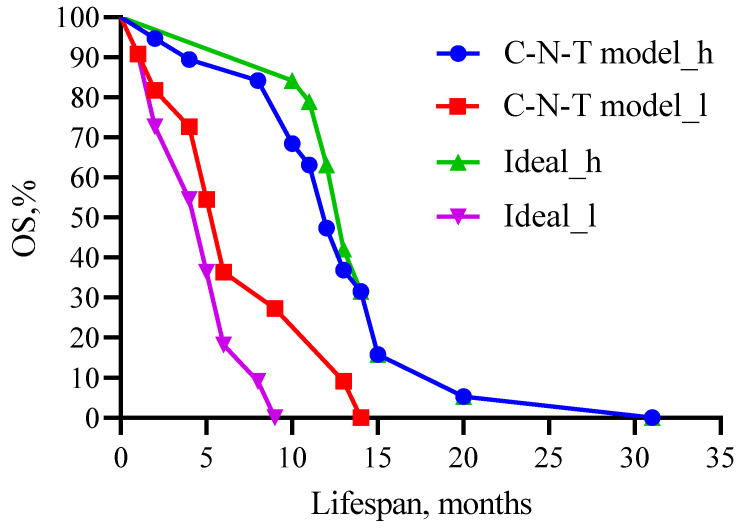
Comparison of patient survival prognosis in the predictive model with the theoretical (ideal) model for CARB, band neutrophils, and total protein as predictors and their threshold values. C—carboplatin; N—neutrophil; T—total protein. Threshold values: CARB 4115 μg/mL, NEUT-B 2.5%, and TP 64.5 g/L. C-N-T—whole model carboplatin-band neutrophils-total protein.

We performed multivariate Cox regression analysis to compare the survival probabilities of patients in the predictive model for carboplatin, band neutrophils, and total protein as predictors and their cut-off values with the theoretical (ideal) model (Figure 11).

Figure 11 shows that no statistical difference was observed when comparing high- (χ^2^ = 0.24, 12 versus 13 months, *p* = 0.6242) and low-life-expectancy groups (χ^2^ =1.341, 6 versus 5 months, *p* = 0.2469) between the predictive and theoretical model.

To study the interaction of factors with each other and their influence on the survival and life expectancy of GBM patients, we performed a two-way ANOVA and the Sobel test. The results showed that the main factor of the model affecting the life expectancy of patients is the IC_50_ of CARB (*p* < 0.0001). The Sobel test is used to determine the significance of mediation effects in a model. It assesses whether the effect of an independent variable on a dependent variable is mediated by a third variable, known as a mediator (Table 6).

The results of the Sobel test show that there are no interactions between the components of the model, and each component is an independent factor influencing the event (life expectancy, survival) of GBM patients.

## 4. Discussion

In this manuscript, a predictive model for the life expectancy of GBM patients is presented, including the measurement of the IC_50_ of CARB for tumor cells, as well as the levels of band neutrophils and total protein in the patient’s blood. This model, developed with the participation of 31 patients and IC_50_ data for six chemotherapy drugs and 32 blood parameters (cells, proteins) as a tumor microenvironment using statistical ANOVA, ROC analysis, and PCA, allows us, based on all three parameters, to predict the OS of GBM patients with 83.3% probability (sensitivity and specificity). The AUC for our model was 0.889. The results of the model were also confirmed by the Kaplan–Meier analysis of patients’ lifespan. Rikan B.S. et al. evaluated the predictive performance of six regression models, XGBoost, AdaBoost, DT, KNN, RF, and DNN machine learning, to assess the factors affecting the survival of GBM patients [32]. The authors found that age was the main factor affecting the survival of GBM patients in the DNN model with an accuracy of 90.25% and R^2^ 0.6565. Yoon H.G. et al. examined the predictive performance of machine learning models based on clinical data (age, survival time, sex, ECOG performance status, resection, IDH mutation, MGMT hypermethylation, adjuvant TMZ cycles, total radiotherapy dose) from 118 GBM patients who received radiochemotherapy. The concordance index (C-index) and AUC were calculated. The model’s C-index and AUC were 0.768 (95% CI: 0.759, 0.776) and 0.790 (95% CI: 0.783, 0.797 *p* < 0.001), respectively [33]. Recently, Peres N. et al. developed a prognostic model for the survival of GBM patients using a neural network algorithm. In this model, as in ours, positive predictors were tumor microenvironment cells, tumor-associated macrophages (TAMs) and PD-L1 ligands, while increased CD86 expression turned out to be a negative prognostic indicator [34].

Madhugiri V.S. et al. found that absolute eosinophil count and low neutrophil/eosinophil ratio were predictors of increased life expectancy and survival in GBM patients [35]. We also found positive relationships between eosinophil and basophil levels and patient survival (Table 2 and Table 4, Figure 2). Duan X. et al. studied the prognostic significance of the neutrophil-to-lymphocyte ratio (NLR), monocyte-to-lymphocyte ratio (MLR), platelet-to-lymphocyte ratio (PLR), and platelet-to-fibrinogen ratio (FPR) in 281 GBM patients on 3-year survival. One-way Cox regression analysis showed that NLR (HR = 1.456, 95% CI: 1.286–1.649, *p* < 0.001), MLR (HR = 1.272, 95% CI: 1.120–1.649, *p* < 0.001), and FPR (HR = 1.183, 95% CI: 1.049–1.333, *p* < 0.001) were associated with prognosis and OS in GBM. The AUC in the test and validation sets was 0.907 and 0.900, respectively [36]. In our study, under conducting a one-way variance ANOVA analysis, platelets and neutrophils also correlated with the survival of GBM patients (Table 3, Figure 2). Gan Y et al. also found in 113 GBM patients that NLR above or below 3 correlated with patients’ OS of 9.6 and 17.1 months, respectively. These authors also showed in univariate analysis that a preoperative Karnofsky performance score ≥ 80, the extent of tumor resection, adjuvant radiotherapy with TMZ, NLR ≥ 3, and lymphocyte count ≥ 1.6 × 10^9^/L correlated with patients’ OS [37].

Similar to our study, Zheng L. et al. investigated the relationship of eosinophils, basophils, neutrophils, lymphocytes, and monocytes levels with GBM progression-free survival (PFS) in 268 GBM patients. The authors performed Kaplan–Meier analysis and Cox regression and found that basophils ≥ 0.015 × 10^9^/L (*p* = 0.015) and lymphocytes ≥1.555 × 10^9^/L (*p* = 0.005) correlated with better PFS and were independent prognostic factors for PFS. The concordance index (C index) for predicting PFS was 0.629 [38]. Analogically to these results, Yang C. et al., using multivariate Cox regression and Kaplan–Meier analysis, showed that a high platelet-to-basophil ratio (>4575) (HR = 1.819, 95% CI: 1.110–2.980, *p* = 0.018) was associated with lower OS in GBM patients (concordance index = 0.844, AUC = 0.632) [39]. Saito T. et al. studied the number of circulating blood cells in 50 GBM patients, who received TMZ therapy. The researchers assessed the relationship between blood cells (WBCs, neutrophils, lymphocytes, red blood cells, and platelets) during the concomitant TMZ phase and OS of patients. Factors such as age, gender, Karnofsky performance status resection extent, O6-methylguanine-DNA methyltransferase (MGMT) status, the rate of decline in WBCs, neutrophils, and platelets significantly correlated with the OS of patients. Patients with low (less than 40%) neutrophil levels had significantly longer OS than those with high neutrophil levels (more than 40%, HR = 2.815; 95% CI: 1.177–7.038; *p* = 0.0196) [40]. The activation of and increase in lymphocytes’ level indicates an increase in the immune response, which is accompanied by a decrease in the size of the GBM and an increase in the life expectancy of patients [41].

Vaitkiene P et al. used custom human protein antibody arrays, including 10 proteins—angiopoietin-1 (ANGPT1), amphiregulin (AREG), insulin-like growth factor-1 (IGF1), interferon gamma-induced protein 10 (IP10/CXCL10), matrix metalloproteinase-2 (MMP2), neural cell adhesion molecule 1 (NCAM1), osteopontin (OPN), plasminogen activator inhibitor-1 (PAI1), transforming growth factor-beta1 (TGFβ1), and tissue inhibitor-1 of metalloproteinases (TIMP1)—from 59 patients’ samples with astrocytoma and 43 control blood sera. The analysis showed that the level of OPN was a predictor of 12-month survival for GBM patients with a specificity of 84% [42]. Of interest is the recent article by Zhang W et al., in which they studied the impact the genes’ expression of immunosuppressive protein ligand PD-L1 and tumor-infiltrating lymphocyte (TIL) infiltration in the tumor microenvironment on the OS of GBM patients. The metalloreductase *STLEAP3* gene was to be involved in glioma progression. Tumor purity analysis showed that PD-L1 and TIL were positively correlated with OS and negatively correlated with tumor purity (the proportion of cancer cells in the sample) [43].

We show that the use of these three parameters (IC_50_ of CARB, levels of band neutrophils, and total protein) can reflect the integral effectiveness of therapy on the life expectancy of patients. The inclusion of CARB in the final model, in addition to the statistical analysis data, was dictated by its mechanisms of action on tumor cells, the development of their drug resistance, and its pharmacokinetics and toxicity profile, which, in general, affected the survival and lifespan of the patients [44,45]. CARB is known to alkylate DNA nitrogen bases, which disrupts its replication and biosynthesis and induces to the death of cancer cells. After intravenous administration (300–500 mg/m^2^), CARB does not bind to plasma proteins, and its concentration is linearly dependent on the dose of the drug with an average circulation time of 4.4 L/h. An increase in the dose of CARB during phase 1 clinical trials in 10 patients with recurrent malignant gliomas was not accompanied by the development of toxic effects on the organism and the registration of death cases [45]. Low binding of CARB to plasma proteins and its long-term presence in the blood allows this drug to effectively affect tumor cells in the long term, inducing their death. Unlike CARB, the mechanism of action of TMZ is due to methylation of guanine nucleotides of DNA, which is often inhibited by the expression of the O6-methylguanine DNA methyltransferase (MGMT) enzyme, causing the development of drug resistance in GBM cells [46]. In addition to this mechanism, resistance to TMZ is ensured by DNA repair mechanisms like the mismatch repair pathway (MMR) or base excision repair (BER), abnormal signaling pathways, autophagy, epigenetic modifications, microRNAs, and extracellular vesicle production [47]. According to the MTT analysis, among our 31 GBM patients, 24 (77.4%) had high IC_50_ values of TMZ (12535 ± 2136.4), which indicates the development of drug resistance in GBM. In addition, TMZ is a prodrug, the active metabolite of which, monomethyltriazenoimidazolecarboxamide (MTIC), is formed at a physiological pH value; however, the use of TMZ is often accompanied by the development of toxic effects, including nausea, vomiting, edema, leukopenia, lymphopenia, neutropenia, and thrombocytopenia. These effects change the physiological pH of the blood and, therefore, disrupt the conversion of the proform of TMZ into an active cytotoxic drug. Moreover, the pharmacokinetics of TMZ after oral administration are due to rapid absorption and rapid excretion from the organism with urine. The plasma half-life is approximately 1.8 h, and it binds to plasma proteins more strongly than CARB (12–16%) [48,49]. The use of TMZ or an increase in its dose in GBM patients is often accompanied by the development of drug resistance in tumors, which we observed in the MTT assay when determining the IC_50_ of TMZ, and on the other hand, it depends on and is limited by the degree of reduction in the levels of leukocytes, lymphocytes, neutrophils, and platelets in the blood of patients. In general, all these events occurring during the treatment of GBM patients with TMZ can affect their survival and lifespan.

On the other hand, the levels of neutrophils and WBCs in the blood of GBM patients have been identified as prognostic factors for patients’ survival. For example, Wang Z et al., among 288 patients with glioma, found via ROC analysis that WBCs (AUC = 0.676, 95% CI: 0.61–0.741, *p* < 0.0001) and NEUs (AUC = 0.726, 95% CI: 0.663–0.789, *p* < 0.0001) were negative predictive factors for survival in TMZ treatment. At the same time, high levels of WBCs (*p* = 0.00086), NEUs (*p* < 0.0001) were associated with shorter OS in patients receiving TMZ. However, the difference was not statistically significant for GBM patients in the non-TMZ treatment group (PFS: *p* = 0.51; OS: *p* = 0.3) [50]. This study found that it was an increase in band neutrophils, but not segmented (mature) neutrophils, that was associated with a decrease in lifespan in GBM patients. This may be due to the fact that band neutrophils lack azurophilic granules and lysosomes; myeloperoxidase, which produces molecular oxygen from hydrogen peroxide; as well as 5′-nucleotidase, which contribute to the formation of reactive oxygen species (ROS) and destroy cell DNA. Mature neutrophils also contain specific small granules, which include the enzymes lysozyme, alkaline phosphatase, and lactoferrin, as well as NADPH oxidase, which catalyzes the formation of ROS that determine their cytotoxic properties. Segmented neutrophils express G-CSF receptors (the main regulator of neutrophil development), IL-17, IL-23, and the main chemotactic factor IL-8 (CXCR1 and CXCR2 receptors) on the membrane, affecting their development, migration, and activation [51]. All these features of segmented neutrophils, in comparison with band neutrophils, give them advantages in inhibiting the growth of cancer cells. However, segmented neutrophils express the chemokine SDF-1, which through the CXCR4 receptor ensures their interaction with GBM cells and enhances their proliferation, angiogenesis, and invasion into surrounding tissues [52,53]. Azurophilic granules of segmented neutrophils also contain N-acetylglucosaminidase, proteases (cathepsin G, elastase, collagenase), and specific granules contain matrix metalloprotease-9 [51]. All these enzymes are involved in the destruction of the extracellular matrix of tissues and GBM, ensuring the invasion and metastasis of its cells [54,55]. The content of antitumor and tumor-stimulating proteins in segmented neutrophils makes these cells less specific as predictors of patient survival. This conclusion is consistent with our results, as shown in Table 4.

The use of total protein as a component of the final predictive model was determined, on the one hand, by the obtained statistical patterns (Table 3 and Table 4; Figure 2, Figure 4 and Figure 6), and on the other hand, by its composition and functional role in the body, including in the development of cancer. It is known that the level of serum albumin, which makes up 52.9–66.9% of the total protein [56], positively correlates with the survival of patients with glioma. Han S. et al., in 214 GBM patients, found that serum albumin levels were significantly correlated with OS in glioblastoma patients (multivariate HR = 0.966; *p* = 0.023). The authors conducted an ROC analysis that showed that the median level (35.35 g/L) of serum albumin has 62.5% sensitivity and 63.7% specificity for 1-year-shorter versus 1-year-longer survival cases [57]. Multivariate analysis indicated that total protein (HR = 0.978, 95%CI: 0.960–0.998, *p* = 0.029), prealbumin levels (HR = 0.997, 95%CI: 0.993–1.000, *p* = 0.041), and MGMT promoter methylation (HR  = 0.618, 95% CI: 0.387–0.988, *p* = 0.044) were independently associated with OS in GBM patients. Both albumin and total protein levels are known nutritional indicators. These proteins are a reserve for providing the body with the protein it needs: amino acids, cytokines, growth factors, antibodies, receptors, enzymes, peptides with antitumor activity, complex proteins, and phospho-, lipo-, metallo- and nucleoproteins. In univariate analysis, all these nutritional parameters were associated with OS, which demonstrated the prognostic value of these factors. Thus, the prognostic effect of serum albumin levels may be partly due to its role as a nutritional parameter [57]. In addition, Han S et al. showed that low serum albumin levels are associated with higher IGFBP-2 levels, which inversely correlates with OS in patients with glioblastoma [58,59]. A high concentration of exogenous IGFBP-2 stimulates proliferation, invasion, and chemoresistance to TMZ in GBM cells via the β1-ERK integrin pathway [58,59]. Moreover, blood–brain barrier permeability may be higher in GBM patients with low serum albumin levels [60]. Thus, low serum albumin levels associated with high serum IGFBP-2 levels and blood–brain barrier disruption may result in poor survival. In addition, GBM cells may induce inflammatory responses [61]. In inflammatory conditions, high serum IGFBP-2 levels are associated with elevated levels of the cytokine interleukin (IL)-6 [62], another prognostic factor for GBM [63], which negatively regulates serum albumin levels by increasing catabolism and downregulating synthesis in the liver, further worsening the nutritional status of the patient. Thus, during inflammatory reactions caused by GBM, the interaction between albumin, IGFBP-2, and IL-6 may significantly affect the pancreas of patients. The second most abundant fraction of total protein after albumin is gamma globulins (19.2%) [55], which include, among others, antiviral and tumor-specific antibodies [64]. Levels of these antibodies were associated with the risk of developing glioma among 197 Swedish patients [64]. Moreover, these parameters (band neutrophil count and total protein) are generally available, fast, and carried out everywhere in any clinic. To determine the IC_50_ of CARB, a tumor biopsy material and a culture laboratory are required. The MTT assay is also a fairly fast and common test, which generally makes the use of this model realistic in clinical conditions.

The present study had several limitations. First, the variability that may exist between different laboratories in determining the IC_50_ of CARB and the lack of uniform standards may affect the accuracy (sensitivity and specificity, drug cutoff levels) of the model. Second, the lack of serial dynamic levels of total protein and band neutrophils is another limitation. Third, the retrospective design of the study may lead to changes in the parameters and their absolute values during clinical trials. Fourth, the determination of IC_50_ requires obtaining a cell culture of GBM, which is time-consuming (several days). This time delay may negatively affect clinical decision-making when choosing a drug, dosing regimen, and treatment protocol for a group of sensitive patients, based on the model results and who requires immediate therapy due to tumor progression. Fifth, the number of cases that completed therapy was not large enough (n = 31), of which 24 (77.4%) individuals had tumor cells that showed high IC_50_ values for TMZ (12,535 ± 2136.4), which indicates the development of drug resistance in tumor cells and limits the power of this study. Sixth, GBM is a highly heterogeneous tumor, both in cellular composition and at the molecular genetic level, and these characteristics affect patient survival, which, given their small sample, can affect the lifespan rates and model parameters. However, in addition to the MTT assays, this article included histological, immunohistochemical, clinical, and biochemical studies. Histological and immunohistochemical analyses were necessary to verify the histological diagnosis of GBM in contrast to other brain tumors and determine its type (IDH1+, IDH1−).

When using a prognostic model in clinical practice, it is necessary to take into account the above limitations, as well as the patient’s health status and a set of clinical and biochemical blood parameters reflecting the functions of the liver, kidneys, and immune and nervous systems. If, for any reason, deviations from the model prognosis are observed in a real situation, it is necessary, if possible, to establish and eliminate the cause that caused these changes and adjust tumor therapy by reducing or increasing the doses of chemotherapy drugs, replacing or discontinuing chemotherapy drugs, and prescribing concomitant anti-inflammatory, bacterial, or antiviral therapy in accordance with currently accepted international clinical standards [65,66]. It is also necessary to take into account the dynamics of changes in hematological and biochemical parameters in a patient, so it is necessary to focus on more stable indicators, which can lead to the replacement of some indicators in the model with others with a similar function. For example, total protein in case of hyper- or hypoproteinemia can be replaced with albumin. Band neutrophils, when their level is unstable or there is neutrophilia or neutropenia, are divided into total or segmented neutrophils.

## 5. Conclusions

Based on the in vitro GBM cells’ sensitivity of patients to chemotherapy drugs and NGF, as well as the levels of blood cells and proteins that are part of the tumor microenvironment, a simple predictive model for assessing the life expectancy of GBM patients has been developed using statistical analysis methods. This model includes an assessment of only three parameters: an IC_50_ of CARB higher 4115 μg/mL in tumor cells, as well as levels of band neutrophils below 2.5% and total protein above 64.5 g/L in the blood, which allows predicting with 83.3% probability the life expectancy of patients by 15 months or more. This will allow physicians to personalize and adjust therapy protocols for cancer patients, assessing their health status dynamically during treatment courses and thereby increasing the therapy effectiveness and the life expectancy of the patients. In other words, this study identifies indicators and their cutoff values that will allow physicians to evaluate the effectiveness of therapy that affects the patient’s life expectancy. However, future confirmatory studies using other platforms are needed to prove the effect of drugs on GBM cells, including PCR, immunohistochemical analysis, and fluorescence microscopy. The developed model needs to be validated on a larger patient sample and in clinical practice.

## Figures and Tables

**Table 1:** Doses of NGF and chemotherapy.

Drugs	Dose, μM
Doxorubicin	920, 460, 230, 115, 73.6, 36.8, 18.4
Carboplatin	26,900, 2690, 1350, 673, 269, 134
Cisplatin	1660, 830, 332, 166, 83, 33.2, 16.1
Temozolomide	15,500, 5150, 1550, 773, 386, 155
Etoposide	27, 13.5, 6.7, 3.3, 1.6, 0.8
NGF	0.2, 0.1, 0.05, 0.025, 0.0125, 0.006

NGF, chemotherapeutic drugs were studied using nonlinear regression analysis with Origin Pro 8.5 program.

**Table 2:** Clinical and molecular subtype of GBM, lifespan of patients, and IC_50_ of chemotherapeutic agents and NGF.

ID Patient	Gender, m, f	Age, y	Molecular Subtype	Ki-67, %	Lifespan, Months	IC_50_, μM					
						DOX	CARB	TMZ	CIS	ETO	NGF
11081	m	74	wt	23–30	2	290.4	29,431	16,179.5	2448.4	27.0	>0.006
11961	m	59	wt	10–12	10	3350.3	4000.0	43,539.3	11,919.7	86.5	0.029
6770	m	74	wt	18–25	8	850.0	2000.0	14,000.0	1090.0	26.3	0.007
7934	m	63	wt	18–20	6	50.9	888.8	7491.0	200.0	7.5	>0.006
49142	m	63	wt	8–10	20	548.3	3093.6	11,056.0	776.0	11.4	0.007
25873	f	61	NOS		1	560.0	2708.4	8619.2	300.0	8.9	>0.006
57595	f	67	wt	15–20	6	16.9	888.8	194.5	1682.3	7.5	>0.006
55068	m	66	NES	25–30	14	546.5	39,792.9	4789.5	1104.8	11.8	>0.006
15159	m	61	NOS		4	179.2	27,574.5	436.8	698.1	11.4	0.016
62642	f	36	wt	35–40	12	20.3	116.4	24,015.7	1158.5	32.3	0.008
60886	m	73	NOS, NES		11	278.8	42,495.1	2174.3	>1660.0	6.3	0.007
18871	f	52	wt	20		2682.8	20,852.7	11,976.9	1776.4	30.9	0.009
114495	f	72	wt		4	3040.0	20,471.8	40,009.3	965.8	40.0	0.016
10677	m	44	wt	40	5	1180.1	4498.0	1309.1	2448.4	3.4	>0.006
1401	m	63	NOS		31	920.0	20,471.8	611.8	261.2	10.3	0.008
18871	f	52	wt	20	15	2682.8	30.9	11,976.9	1776.3	30.9	0.008
8989	m	31	wt	50–70	2	817.1	5136.5	14,486.0	1218.8	26.3	0.006
20939	m	53	wt	30–35	15	900.0	24,031.9	15,500.0	476.5	38.0	>0.006
39114	m	75	wt	20–25	10	3458.6	20,195.2	12,282.1	1824.2	32.8	>0.006
40906	f	66	wt	25–30	13	1083.2	17,861.9	14,961.7	1596.1	58.9	>0.006
48993	m	63	wt	8–10	2	-	1126.8	15,407.5	120.4	38.7	>0.006
48307	m	44	wt	8–12		1260.3	38,147.6	1510.7	1280,8	9.5	0.038
9439	f	55	wt	40–50	13	1513.2	1126.8	22,206.3	1784.9	41.3	0.011
10448	f	73	wt	20–25	14	478.7	20,852.7	14,659.1	1299.0	3.4	0.66
27980	m	51	wt	25	13	733.4	26,116.5	5345.6	835.3	9.3	>0.006
12645	m	66	wt	35–40	12	483.6	24,237.2	5258.3	729.8	7.0	>0.006
7593	m	59	wt	15–20	5	1123.9	2223.4	14,905.5	298.9	26.8	0.027
13275	f	39	wt	35–40	12	870.0	4605.4	17,700.0	770.5	18.0	>0.006
121509	f	71	wt	10–15	15	56.7	2110.4	1900.0	1880.0	8.5	>0.006
13447	m	52	wt	3–5	9	440.0		15,000.0	640.0	10.4	>0.006
65829	m	57	wt	15	13	0.0001	2421.4	13,605.3	936.4	8.5	>0.006

Note: m—male; f—female; y—years; wt—IDH1 wild type; NOS—not otherwise specified; nestin—NES.

**Table 3:** Associations of parameters with the life expectancy of GBM patients using ANOVA.

Parameter	Mean Diff	95.00% CI of Diff	Significant	Adjusted *p* Value
IC_50_ Doxorubicin	−903.8	−1885 to 77.35	No	0.081322
**IC_50_ Carboplatin**	**−16,532.0**	**−30,023 to −3041**	Yes, *	**0.011710**
**IC_50_ Temozolomide**	**−7988.0**	**−14,583 to −1393**	Yes, *	**0.012839**
**IC_50_ Cisplatin**	**−1170.0**	**−1899 to −440.5**	Yes, **	**0.001065**
IC_50_ Etoposide	−7.889	−23.84 to 8.062	No	0.727249
**IC_50_ NGF**	**10.33**	**3.314 to 17.34**	Yes, **	**0.003418**
WBC	−2.833	−13.11 to 7.442	No	0.994316
RBC	6.117	−1.429 to 13.66	No	0.160468
**PLT**	**−254.2**	**−328.0 to −180.4**	Yes, ****	**<0.0001**
LYM, %	−5.140	−13.33 to 3.053	No	0.428481
LYM	−7.067	−15.57 to 1.432	No	0.141795
**NEUT, %**	**−64.01**	**−79.85 to −48.18**	Yes, ****	**<0.0001**
**NEUT-B**	**7.619**	**0.6224 to 14.62**	Yes, *	**0.028720**
**NEUN-S**	**−60.87**	**−76.12 to −45.61**	Yes, ****	**<0.0001**
MON	3.333	−4.390 to 11.06	No	0.858937
**IMG**	**9.147**	**1.437 to 16.86**	Yes, *	**0.015081**
**MC**	**9.417**	**2.268 to 16.57**	Yes, **	**0.008010**
**MMC**	**9.970**	**2.580 to 17.36**	Yes,**	**0.007362**
**BAS, %**	**10.12**	**2.338 to 17.90**	Yes, **	**0.007255**
**BAS**	**10.13**	**2.273 to 17.99**	Yes, **	**0.007789**
**EOS, %**	**9.647**	**1.884 to 17.41**	Yes, *	**0.010474**
**EOS**	**9.687**	**1.975 to 17.40**	Yes, **	**0.009620**
**Hb**	**−112.5**	**−124.9 to −100.0**	Yes, ****	**<0.0001**
AST	−12.47	−33.17 to 8.234	No	0.454599
ALT	−46.87	−96.68 to 2.935	No	0.071985
BUN	0.004167	−9.107 to 9.115	No	>0.9999
**ALB**	**−29.83**	**−38.53 to −21.14**	Yes, ****	**<0.0001**
**TP**	**−53.89**	**−62.03 to −45.74**	Yes, ****	**<0.0001**
CRB	−6.203	−23.24 to 10.83	No	0.954377
**D-dimer**	**−771.7**	**−1279 to −264.1**	Yes, **	**0.004318**
**HCT**	**−24.24**	**−31.37 to −17.11**	Yes, ****	**<0.0001**
**PCT**	**10.09**	**2.324 to 17.85**	Yes, **	**0.007293**
UREA	2.928	−7.432 to 13.29	No	0.988580
GLU	3.580	−4.736 to 11.90	No	0.835504
**CREAT**	**−61.90**	**−79.32 to −44.49**	Yes, ****	**<0.0001**
FBN	7.028	−1.515 to 15.57	No	0.143016
**GFR**	**−113.9**	**−162.0 to −65.70**	Yes, ****	**<0.0001**
**APTT**	**−14.79**	**−22.79 to −6.802**	Yes, ***	**0.000634**

Note: Statistically significant differences are highlighted in bold. ALB—albumin; ALT—alanine aminotransferase; APTT—activated partial thromboplastin time; AST—aspartate aminotransferase; BAS—basophil; BUN—total bilirubin; CRB—C-reactive protein; CREAT—creatinine; EOS—eosinophil; FBN—fibrinogen; GFR—glomerular filtration rate; GLU—glucose; Hb—hemoglobin; HCT—hemocrit; IMG—immature granulocytes; LYM—lymphocyte; MMC—metamyelocyte; MON—monocyte; MC—myelocyte; NEUT—neutrophil; PLT—platelet; PCT—plateletcrit; RBC—red blood cell; TP—total protein; WBC—white blood cell. Blood samples were taken within 3 days prior to surgery from all patients with GBM. Signs *, **, ***, **** indicate statistically significant differences at *p* < 0.05, *p* < 0.01, *p* < 0.001, *p* < 0.0001, respectively.

**Table 4:** Calculation of AUC, sensitivity, and specificity of survival of GBM patients’ predictors using ROC analysis.

Parameter	Threshold Value	AUC	Sensitivity, %	Specificity, %	Likelihood Ratio	*p* Value
Doxorubicin	<418.5	**0.7600**	80.00%(95% CI 37.55–98.97%)	80.00% (95% CI 37.55–98.97%)	4.0	0.1745
Carboplatin	<4115	**0.8000**	80.00% (95% CI 37.55–98.97%)	80.00% (95% CI 37.55–98.97%)	4.0	0.1172
Temozolomide	<9838	0.6400	80.00% (95% CI 37.55–98.97%)	60.00% (95% CI 23.07–92.89%)	2.0	0.4647
Cisplatin	<737.1	0.5200	60.00% (95% CI 23.07–92.89%)	80.00% (95% CI 37.55–98.97%)	3.0	0.9168
Etoposide	<10.57	**0.8200**	60.00% (95% CI 23.07–92.89%)	80.00% (95% CI 37.55–98.97%)	3.0	0.0947
NGF	<0.002900	0.6750	60.00% (95% CI 23.07–92.89%)	75.00% (95% CI 30.06–98.72%)	2.4	0.3913
WBC	>8.6	0.6458	83.33% (95% CI 43.65–99.15%)	46.67% (95% CI 24.81–69.88%)	1.563	0.1296
RBC	>3.935	0.5000	87.50% (95% CI 52.91–99.36%)	57.14% (95% CI 25.05–84.18%)	2.042	>0.9999
Platelets	<288.0	0.5179	71.43% (95% CI 35.89–94.92%)	37.50% (95% CI 13.68–69.43%)	1.143	0.9079
Lymphocytes, %	<13.80	**0.7500**	66.67% (95% CI 30.00–94.08%)	75.00% (95% CI 40.93–95.56%)	2.667	0.1213
Lymphocytes	<18.50	**0.7653**	71.43% (95% CI 35.89–94.92%)	71.43% (95% CI 35.89–94.92%)	2.5	0.0967
Neutrophils, %	>72.20	0.6786	62.50% (95% CI 30.57–86.32%)	57.14% (95% CI 25.05–84.18%)	1.458	0.2472
**Band neutrophils**	**>2.500**	**0.9063**	**87.50% (95% CI 52.91–99.36%)**	**83.33% (95% CI 43.65–99.15%)**	**5.25**	0.0118 *
Segmented neutrophils	>71.00	0.6518	57.14% (95% CI 25.05–84.18%)	62.50% (95% CI 30.57–86.32%)	1.524	0.3253
Monocytes, %	<7.500	0.6327	71.43% (95% CI 35.89–94.92%)	71.43% (95% CI 35.89–94.92%)	2.50	0.4062
Eosinophils, %	<0.4500	0.6071	100.0% (95% CI 64.57–100.0%)	37.50% (95% CI 13.68–69.43%)	1.6	0.4875
Basophils, %	<0.1500	0.6071	71.43%(95% CI 35.89–94.92%)	50.00%(95% CI 21.52–78.48%)	1.429	0.4875
Myelocytes	>0.5000	0.5952	57.14% (95% CI 25.05–84.18%)	66.67% (95% CI 30.00–94.08%)	1.714	0.5677
Hemoglobin	<124.5	0.6339	57.14% (95% CI 25.05–84.18%)	50.00% (95% CI 21.52–78.48%)	1.143	0.3854
ALT	>40.43	**0.7619**	71.43% (95% CI 35.89–94.92%)	66.67% (95% CI 30.00–94.08%)	2.143	0.1161
AST	>16.89	**0.8286**	71.43% (95% CI 35.89–94.92%)	80.00% (95% CI 37.55–98.97%)	3.571	0.0618
Total bilirubin	<8.435	0.6857	57.14% (95% CI 25.05–84.18%)	80.00% (95% CI 37.55–98.97%)	2.857	0.2912
Albumin	<39.18	**0.8000**	71.43% (95% CI 35.89–94.92%)	80.00% (95% CI 37.55–98.97%)	3.571	0.0882
Total protein	<64.65	**0.7857**	71.43% (95% CI 35.89–94.92%)	83.33%(95% CI 43.65–99.15%)	4.286	0.0865
CRB	>5.150	0.5536	75.00%(95% CI 30.06–98.72%)	14.29% (95% CI 7.32–51.31%)	0.875	0.7285
D-dimer	<489.6	0.6000	40.00% (95% CI 7.10–76.93%)	75.00% (95% CI 40.93–95.56%	1.60	0.6242
Fibrinogen	<2.805	0.6429	50.00% (95% CI 18.76–81.24%)	85.71% (95% CI 48.69–99.27%)	3.50	0.3914
Hematocrit	<34.45	0.6122	57.14% (95% CI 25.05–84.18%)	57.14% (95% CI 25.05–84.18%)	1.333	0.4822
Plateletcrit	<0.2350	0.6667	66.67% (95% CI 30.00–94.08%)	75.00% (95% CI 40.93–95.56%)	2.667	0.3017
Creatinine	>72.98	0.6327	57.14% (95% CI 25.05–84.18%)	71.43% (95% CI 35.89–94.92%)	2.00	0.4062
GFR	>101.6	0.5429	60.00% (95% CI 23.07–92.89%)	42.86% (95% CI 15.82–74.95%)	1.05	0.8075
APTT	<25.60	**0.7500**	100.0% (95% CI 51.01–100.0%)	57.14% (95% CI 25.05–84.18%)	2.333	0.1859

Note: Statistically significant differences and AUC higher than 0.750 are highlighted in bold. Blood samples were taken within 3 days prior to surgery from all patients with GBM. Sign * indicates statistically significant differences at *p* < 0.05.

**Table 5:** Impact of indicators on lifespan of GBM patients by R squared.

Parameter	R^2^	Adjusted *p* Value
**Doxorubicin**	**−0.2810**	**0.0161**
**Carboplatin**	**0.5918**	**0.0066**
Temozolomide	−0.2459	0.9145
**Cisplatin**	**−0.6784**	**0.0024**
Etoposide	−0.1939	0.2406
**NGF**	**−0.7353**	**0.0074**
WBC	−1.617	0.8918
RBC	0.2629	0.3670
Platelets	0.0724	0.8899
Lymphocytes	−3.004	0.4452
Lymphocytes, %	−2.470	0.4462
Neutrophils, %	−0.2826	0.6512
Band neutrophils	−4.566	0.3806
Segmented neutrophils	−0.6154	0.4286
Monocytes, %	−6.727	0.1631
**Eosinophils, %**	**−0.6002**	**0.0004**
**Basophils, %**	**−1.041**	**0.0010**
**Myelocytes, %**	**−0.2986**	**0.0001**
Hemoglobin	0.2803	0.5614
**ALT**	**0.000**	**0.0096**
**AST**	**0.007348**	**<0.0001**
Total bilirubin	0.07651	0.1118
Albumin	0.1561	0.1550
**Total protein**	**0.2067**	**0.0506**
**C-reactive protein**	**0.002105**	**0.0023**
D-dimer	0.05458	0.2427
Fibrinogen	0.0003714	0.2324
Hematocrit	0.2083	0.4492
Plateletcrit	0.2739	0.1970
Creatinine	0.04685	0.8949
Glomerular filtration rate	0.02162	0.2649
APTT	0.2988	0.5983

Note: Statistically significant differences are highlighted in bold.

**Table 6:** Sobel test values are significance levels for factors and mediators of the predictive model.

Factors/Mediators	Carboptatin	Band Neutrophils	Total Protein
Carboplatin	-	−0.128, *p* = 0.448, *p* = 0.897	0.974, *p* = 0.164, *p* = 0.329
Band Neutrophils	−0.128, *p* = 0.448, *p* = 0.897	-	0.129, *p* = 0.448, *p* = 0.896
Total protein	0.974, *p* = 0.164, *p* = 0.329	0.129. *p* = 0.448, *p* = 0.896	-

Note: the first of the three numbers is the Sobel test value; the second and third are the one-tailed and two-tailed probabilities, respectively.

## Data Availability

The data presented in this study are openly available on Table S1. Share at https://doi.org/10.6084/m9.figshare.16879432.

## Supplementary Materials

The following supporting information can be downloaded at: https://www.mdpi.com/article/10.3390/biomedicines13051040/s1.

## References

[1] Luo C., Song K., Wu S., Farrukh Hameed N.U., Kudulaiti N., Xue H., Qint Z.-Y., Wu J.-S. (2021). The prognosis of glioblastoma: A large, multifactorial study. Br. J. Neurosurg..

[2] Grochans S., Cybulska A.M., Siminska D., Korbecki J., Kojder K., Chlubek D., Baranowska-Bosiacka I. (2022). Epidemiology of glioblastoma multiforme—Literature review. Cancers.

[3] Rončević A., Koruga N., Koruga A.S., Rončević R., Rotim T., Šimundić T., Kretić D., Perić M., Turk T., Štimac D. (2023). Personalized Treatment of Glioblastoma: Current State and Future Perspective. Biomedicines.

[4] Rice T., Lachance D.H., Molinaro A.M., Eckel-Passow J.E., Walsh K.M., Barnholtz-Sloan J., Ostrom Q.T., Francis S.S., Wiemels J., Jenkins R.B. (2016). Understanding inherited genetic risk of adult glioma—A review. Neurooncol. Pract..

[5] Ivanov S.D. (2019). The influence of non-ionizing radiation on tumor formation in humans and animals. Donosology Healthy Lifestyle.

[6] Alexander B.M., Cloughesy T.F. (2017). Adult Glioblastoma. J. Clin. Oncol..

[7] Seliger C., Meyer A.-L., Leidgens V., Rauer L., Moeckel S., Jachnik B., Proske J., Dettmer K., Rothhammer-Hampl T., Kaulen L.D. (2022). Metabolic Heterogeneity of Brain Tumor Cells of Proneural and Mesenchymal Origin. Int. J. Mol. Sci..

[8] Tam S.Y., Wu V.W.C., Law H.K.W. (2020). Hypoxia-Induced Epithelial-Mesenchymal Transition in Cancers: HIF-1α and Beyond. Front. Oncol..

[9] Dymova M.A., Kuligina E.V., Richter V.A. (2021). Molecular Mechanisms of Drug Resistance in Glioblastoma. Int. J. Mol. Sci..

[10] Su J., Cai M., Li W., Hou B., He H., Ling C., Huang T., Liu H., Guo Y. (2016). Molecularly Targeted Drugs Plus Radiotherapy and Temozolomide Treatment for Newly Diagnosed Glioblastoma: A Meta-Analysis and Systematic Review. Oncol. Res..

[11] Ivanov S.D., Korytova L.I. (2013). Predictive Markers of the Effectiveness of Radiation and Chemoradiotherapy in Oncology.

[12] Jeon J., Lee S., Kim H., Kang H., Youn H., Jo S., Youn B.H., Kim H.Y. (2021). Revisiting platinum-based anticancer drugs to overcome gliomas. Int. J. Mol. Sci..

[13] Leonard A., Wolff J.E. (2013). Etoposide improves survival in high-grade glioma: A meta-analysis. Anticancer Res..

[14] Norouzi M., Yathindranath V., Thliveris J.A., Kopec B.M., Siahaan T.J., Miller D.W. (2020). Doxorubicin-loaded iron oxide nanoparticles for glioblastoma therapy: A combinational approach for enhanced delivery of nanoparticles. Sci. Rep..

[15] Gaglio D., Bianco M.R., Aprea F., Virtuoso A., Bonanomi M., Alberghina L., Papa M., Colangelo A.M. (2018). Differentiation by nerve growth factor (NGF) involves mechanisms of crosstalk between energy homeostasis and mitochondrial remodeling. Cell Death Dis..

[16] Vaishnavi A., Le A.T., Doebele R.C. (2015). TRKing down an old oncogene in a new era of targeted therapy. Cancer Discov..

[17] Zha K., Yang Y., Tian G., Sun Z., Yang Z., Li X., Sui X., Liu S., Zhao J., Guo Q. (2021). Nerve growth factor (NGF) and NGF receptors in mesenchymal stem/stromal cells: Impact on potential therapies. Stem Cells Transl. Med..

[18] Tacconelli A., Farina A.R., Cappabianca L., Desantis G., Tessitore A., Vetuschi A., Sferra R., Rucci N., Argenti B., Screpanti I. (2004). TrkA alternative splicing: A regulated tumor-promoting switch in human neuroblastoma. Cancer Cell.

[19] Maher S., Wynne K., Zhernovkov V., Halasz M. (2024). A temporal (phospho-)proteomic dataset of neurotrophic receptor tyrosine kinase signalling in neuroblastoma. Sci. Data.

[20] Bassili M., Birman E., Schor N.F., Saragovi H.U. (2010). Differential roles of Trk and p75 neurotrophin receptors in tumorigenesis and chemoresistance ex vivo and in vivo. Cancer Chemother. Pharmacol..

[21] Minnone G., De Benedetti F., Bracci-Laudiero L. (2017). NGF and Its Receptors in the Regulation of Inflammatory Response. Int. J. Mol. Sci..

[22] Freshney R.I., Griffiths B., Hay R.J., Reid Y.A., Carmiol S., Kunz-Schugart L., Masters J.R.W. (2000). Animal Cell Culture: A Practical Approach.

[23] Amini S., White M.K. (2013). Neuronal Cell Culture. Methods and Protocols.

[24] Riss T.L., Moravec R.A., Niles A.L., Duellman S., Benink H.A., Worzella T.J., Minor L. (2013). Assay Guidance Manual. Cell Viability Assays.

[25] Pauli D., Seyfarth M., Dibbelt L. (2005). The Abbott Architect c8000: Analytical performance and productivity characteristics of a new analyzer applied to general chemistry testing. Clin. Lab..

[26] Diagnostica Stago S.A.S. (2017). Reagent STA^®^—Cephascreen^®^.

[27] Principal Component Analysis Calculator. https://www.statskingdom.com/pca-calculator.html.

[28] Hu N., Cheng H., Zhang K., Jensen R. (2017). Evaluating the Prognostic Accuracy of Biomarkers for Glioblastoma Multiforme Using the Cancer Genome Atlas Data. Cancer Inform..

[29] van Belle G., Fisher L.D., Heagerty P.J., Lumley T., Fisher L.D., van Belle G. (2004). Biostatistics: A Methodology for the Health Sciences.

[30] Sobel Test Calculator. https://www.danielsoper.com/statcalc/calculator.aspx?id=31.

[31] Chernov A.N., Skliar S.S., Kim A.V., Tsapieva A., Pyurveev S.S., Filatenkova T.A., Matsko M.V., Ivanov S.D., Shamova O.V., Suvorov A.N. (2024). Glioblastoma multiforme: Sensitivity to antimicrobial peptides LL-37 and PG-1, and their combination with chemotherapy for predicting the overall survival of patients. Pharmaceutics.

[32] Rikan B.S., Azar A.S., Naemi A., Mohasefi J.B., Pirnejad H., Wiil U.K. (2024). Survival prediction of glioblastoma patients using modern deep learning and machine learning techniques. Sci. Rep..

[33] Yoon H.G., Cheon W., Jeong S.W., Kim H.S., Kim K., Nam H., Han Y., Lim D.H. (2020). Multi-Parametric Deep Learning Model for Prediction of Overall Survival after Postoperative Concurrent Chemoradiotherapy in Glioblastoma Patients. Cancers.

[34] Peres N., Lepski G.A., Fogolin C.S., Evangelista G.C.M., Flatow E.A., de Oliveira J.V., Pinho M.P., Bergami-Santos P.C., Barbuto J.A.M. (2024). Profiling of Tumor-Infiltrating Immune Cells and Their Impact on Survival in Glioblastoma Patients Undergoing Immunotherapy with Dendritic Cells. Int. J. Mol. Sci..

[35] Madhugiri V.S., Venkatesan S., Dutt A., Moiyadi A.V., Shetty P., Gupta T., Epari S., Jalali R., Sasidharan G.M., Kumar V.R.R. (2023). An Analysis of Eosinophil- and Basophil-Based Indices in Patients with Glioblastoma and their Correlation with Survival. World Neurosurg..

[36] Duan X., Yang B., Zhao C., Tie B., Cao L., Gao Y. (2023). Prognostic value of preoperative hematological markers in patients with glioblastoma multiforme and construction of random survival forest model. BMC Cancer.

[37] Gan Y., Zhou X., Niu X., Li J., Wang T., Zhang H., Yang Y., Liu Y., Mao Q. (2019). Neutrophil/Lymphocyte Ratio Is an Independent Prognostic Factor in Elderly Patients with High-Grade Gliomas. World Neurosurg..

[38] Zheng L., Yu M., Zhang S. (2021). Prognostic value of pretreatment circulating basophils in patients with glioblastoma. Neurosurg. Rev..

[39] Yang C., Xu J., Wang J., Li Z., Yao Q. (2023). Prognostic value of platelet-to-basophil ratio (PBR) in patients with primary glioblastoma. Medicine.

[40] Saito T., Sugiyama K., Hama S., Yamasaki F., Takayasu T., Nosaka R., Muragaki Y., Kawamata T., Kurisu K. (2018). Prognostic importance of temozolomide-induced neutropenia in glioblastoma, IDH-wildtype patients. Neurosurg. Rev..

[41] Sklyar S.S., Trashkov A.P., Matsko M.V., Safarov B.I., Vasiliev A.G. (2022). Immune response to primary glioblastoma. Pediatrician.

[42] Vaitkiene P., Urbanaviciute R., Grigas P., Steponaitis G., Tamasauskas A., Skiriutė D. (2019). Identification of Astrocytoma Blood Serum Protein Profile. Cells.

[43] Zhang W., Liu L., Liu X., Han C., Li Q. (2024). The levels of immunosuppressive checkpoint protein PD-L1 and tumor-infiltrating lymphocytes were integrated to reveal the glioma tumor microenvironment. Environ. Toxicol..

[44] Mrugala M.M., Crew L.K., Fink J.R., Spence A.M. (2012). Carboplatin and bevacizumab for recurrent malignant glioma. Oncol. Lett..

[45] Wang J.L., Barth R.F., Cavaliere R., Puduvalli V.K., Giglio P., Lonser R.R., Elder J.B. (2020). Phase I trial of intracerebral convection-enhanced delivery of carboplatin for treatment of recurrent high-grade gliomas. PLoS ONE.

[46] Lee S.Y. (2016). Temozolomide resistance in glioblastoma multiforme. Genes Dis..

[47] Singh N., Miner A., Hennis L., Mittal S. (2021). Mechanisms of temozolomide resistance in glioblastoma—A comprehensive review. Cancer Drug Resist..

[48] Zhou Q., Guo P., Wang X., Nuthalapati S., Gallo J.M. (2007). Preclinical Pharmacokinetic and Pharmacodynamic Evaluation of Metronomic and Conventional Temozolomide Dosing Regimens. J. Pharmacol. Exp. Ther..

[49] Jezierzański M., Nafalska N., Stopyra M., Furgoł T., Miciak M., Kabut J., Gisterek-Grocholska I. (2024). Temozolomide (TMZ) in the Treatment of Glioblastoma Multiforme—A Literature Review and Clinical Outcomes. Curr. Oncol..

[50] Wang Z., Zhong L., Li G., Huang R., Wang Q., Wang Z., Zhang C., Chen B., Jiang T., Zhang W. (2020). Pre-treatment neutrophils count as a prognostic marker to predict chemotherapeutic response and survival outcomes in glioma: A single-center analysis of 288 cases. Am. J. Transl. Res..

[51] Rosales C. (2018). Neutrophil: A Cell with Many Roles in Inflammation or Several Cell Types?. Front. Physiol..

[52] Wang S.C., Hong J.H., Hsueh C., Chiang C.-S. (2012). Tumor-secreted SDF-1 promotes glioma invasiveness and TAM tropism toward hypoxia in a murine astrocytoma model. Lab. Investig..

[53] Bajetto A., Barbieri F., Dorcaratto A., Barbero S., Daga A., Porcile C., Ravetti J.L., Zona G., Spaziante R., Corte G. (2006). Expression of CXC chemokine receptors 1–5 and their ligands in human glioma tissues: Role of CXCR4 and SDF1 in glioma cell proliferation and migration. Neurochem. Intern..

[54] Klein T., Bischoff R. (2011). Physiology and pathophysiology of matrix metalloproteases. Amino Acids.

[55] Tan G.-J., Peng Z.-K., Lu J.-P., Tang F.-Q. (2013). Cathepsins mediate tumor metastasis. World J. Biol. Chem..

[56] Walker H.K., Hall W.D., Hurst J.W. (1990). Clinical Methods: The History, Physical, and Laboratory Examinations.

[57] Han S., Huang Y., Li Z., Hou H., Wu A. (2015). The prognostic role of preoperative serum albumin levels in glioblastoma patients. BMC Cancer.

[58] Han S., Li Z., Master L.M., Master Z.W., Wu A. (2014). Exogenous IGFBP-2 promotes proliferation, invasion, and chemoresistance to temozolomide in glioma cells via the integrin beta1-ERK pathway. Br. J. Cancer.

[59] Han S., Meng L., Han S., Wang Y., Wu A. (2014). Plasma IGFBP-2 levels after postoperative combined radiotherapy and chemotherapy predict prognosis in elderly glioblastoma patients. PLoS ONE.

[60] Schwartzbaum J.A., Lal P., Evanoff W., Mamrak S., Yates A., Barnett G.H., Goodman J., Fisher J.L. (1999). Presurgical serum albumin levels predict survival time from glioblastoma multiforme. J. Neurooncol..

[61] Borg N., Guilfoyle M.R., Greenberg D.C., Watts C., Thomson S. (2011). Serum albumin and survival in glioblastoma multiforme. J. Neurooncol..

[62] Lo H.C., Tsao L.Y., Hsu W.Y., Chen H.N., Yu W.K., Chi C.Y. (2002). Relation of cord serum levels of growth hormone, insulin-like growth factors, insulin-like growth factor binding proteins, leptin, and interleukin-6 with birth weight, birth length, and head circumference in term and preterm neonates. Nutrition.

[63] Yeung Y.T., McDonald K.L., Grewal T., Munoz L. (2013). Interleukins in glioblastoma pathophysiology: Implications for therapy. Br. J. Pharmacol..

[64] Sjöström S., Hjalmars U., Juto P., Wadell G., Hallmans G., Tjönneland A., Halkjaer J., Manjer J., Almquist M., Melin B.S. (2011). Human immunoglobulin G levels of viruses and associated glioma risk. Cancer Causes Control.

[65] Fernandes C., Costa A., Osório L., Lago R.C., Linhares P., Carvalho B., Caeiro C., De Vleeschouwer S. (2017). Chap.11: Current Standards of Care in Glioblastoma Therapy. Glioblastoma.

[66] Kotecha R., Odia Y., Khosla A.A., Ahluwalia M.S. (2023). Key Clinical Principles in the Management of Glioblastoma. JCO Oncol. Pract..

